# Prevalence and Sociodemographic Correlates of Adolescent Use and Polyuse of Combustible, Vaporized, and Edible Cannabis Products

**DOI:** 10.1001/jamanetworkopen.2018.2765

**Published:** 2018-09-28

**Authors:** Erica N. Peters, Dayoung Bae, Jessica L. Barrington-Trimis, Brantley P. Jarvis, Adam M. Leventhal

**Affiliations:** 1 Battelle Public Health Center for Tobacco Research, Battelle Memorial Institute, Baltimore, Maryland; 2 Center for Family Research, University of Georgia, Athens; 3 Department of Preventive Medicine, Keck School of Medicine of University of Southern California, Los Angeles; 4 University of Southern California Norris Comprehensive Cancer Center, Los Angeles; 5 NorthTide Group, LLC, Edgewood, Maryland; 6 Department of Psychology, University of Southern California, Los Angeles

## Abstract

**Importance:**

Cannabis legalization and commercialization have introduced novel alternative cannabis products, including edible and vaporized cannabis that might appeal to youth and be associated with polyuse (ie, use of ≥2 different products).

**Objective:**

To investigate the prevalence, patterns, and sociodemographic correlates of cannabis product use across combustible, edible, and vaporized administration methods, including polyuse of cannabis via multiple administration methods.

**Design, Setting, and Participants:**

This cross-sectional survey study included 10th-grade students from 10 Los Angeles, California, area high schools from January 2 through October 6, 2015. Students were recruited from respondents in the Happiness and Health Study, an ongoing prospective cohort study in the 10 high schools. Students not in school during administration of the pencil-and-paper survey completed abbreviated surveys by telephone, internet, or mail. Data were analyzed from July 17, 2017, through July 12, 2018.

**Exposures:**

Self-reported sex, race/ethnicity, and socioeconomic status (SES). Students with high SES had parents who attained college or a higher educational level and were ineligible for free or subsidized lunch; those with low SES had parents with lower educational attainment or were eligible for free or subsidized lunch.

**Main Outcomes and Measures:**

Self-report of ever use (yes or no) and past 30-day use (yes or no) status and frequency of use (days in past 30 days) of combustible, edible, and vaporized cannabis.

**Results:**

The sample included 3177 tenth-grade students (93.5% of study enrollees); among these students, 1715 (54.0%) were girls and the mean (SD) age was 16.1 (0.4) years. Among those with available demographic data, respondents were diverse in race/ethnicity (537 [17.2%] Asian; 149 [4.8%] black; 1510 [48.3%] Hispanic; 507 [16.2%] white; 207 [6.6%] multiethnic/multiracial; and 216 [6.9%] other) and included 1654 (60.1%) with a low SES. Prevalence of ever use was highest for combustible products (993 [31.3%]), followed by edible (676 [21.3%]) and vaporized (333 [10.5%]) products. A similar pattern was found for past 30-day use (426 [13.4%] for combustible, 249 [7.8%] for edible, and 156 [4.9%] for vaporized cannabis). Among participants who reported using cannabis in the past 30 days, mean frequency of use of combustible cannabis was higher by 2.65 days (95% CI, 1.40-3.91 days, *P* = .001) than the mean frequency of use for edible cannabis and 1.75 days higher (95% CI, 0.59-2.90 days, *P* = .003) than frequency of use for vaporized cannabis. Most cannabis users (665 [61.7%]) used multiple administration methods, and 260 (8.2%) used all 3 methods. Boys had higher prevalence of ever use for vaporized (170 [11.6%] vs 163 [9.5%]) but not combustible (459 [31.4%] vs 534 [31.1%]) or edible (303 [20.7%] vs 373 [21.7%]) cannabis. Respondents with low SES had higher prevalence of ever use for combustible (614 [37.1%] vs 242 [22.0%]) and edible (408 [24.7%] vs 166 [15.1%]) but not vaporized (186 [11.2%] vs 93 [8.5%]) cannabis.

**Conclusions and Relevance:**

Among 10th-grade students in Los Angeles, use of cannabis via alternative administration methods was of appreciable prevalence, predominately reported in conjunction with other cannabis products and unequally distributed across sociodemographic strata. Prevention programs and regulatory restrictions addressing the spectrum of cannabis products might benefit pediatric public health.

## Key Points

**Question:**

What are the prevalence, patterns, and correlates of use and polyuse of combustible, edible, and vaporized cannabis products among adolescents?

**Findings:**

In a 2015 cross-sectional survey study of 3177 adolescents, ever use of edible (21.3%) or vaporized (10.5%) cannabis was appreciable, and most ever users of cannabis (61.7%) used at least 2 products. Current use was higher in boys than girls for vaporized (6.1% vs 3.0%) but not for combustible (13.8% vs 13.1%) or edible (8.4% vs 7.3%) cannabis.

**Meaning:**

Health professionals should be aware that youth might use a diverse spectrum of combustible and alternative cannabis products.

## Teaser

This cross-sectional survey study investigates the prevalence, patterns, and sociodemographic correlates of cannabis product use and polyuse by administration methods (combustible, vaporized, and edible) among a sample of adolescents in 10th grade.

## Introduction

Adolescent cannabis use poses a substantial public health burden. Adolescent cannabis use is associated with increased risk for chronic cannabis use throughout adulthood, cannabis use disorder, impairment of cognitive development, and lower educational attainment.^1,2,3,4^ In the United States, increasing trends of social normalization of cannabis use, perceptions of reduced harm, and legalization of medical and recreational cannabis use raise new concerns for pediatric health, including an expanding marketplace for novel, noncombustible alternative cannabis products that might attract youth.^5,6,7^

The manufacture of commercially available cannabinoid-infused edibles (eg, gummy bears treated with cannabinoid extracts) and drinks (eg, energy drinks with tetrahydrocannabinol [THC; the principal addictive compound in cannabis]) have diversified the types of ingestible cannabis products that might appeal to adolescents.^6^ Production and marketing of electronic vaporizer devices capable of heating cannabis products to temperatures high enough to aerosolize cannabis without heating beyond the point of combustion (ie, vaping) have also proliferated.^7^ In addition to devices for vaping dry cannabis plant material, the electronic cigarette (e-cigarette) industry has expanded to commercial cannabis products, including the sales of cannabinoid-infused e-cigarette liquids that are available in youth-oriented flavors (eg, bubble gum) and that use marketing strategies that might attract adolescents.^7^

Initial reports^5,8,9,10^ find that appreciable proportions of adolescents use vaporized or edible cannabis products. A systematic study of the prevalence, patterns, and sociodemographic correlates of use and polyuse of alternative cannabis products (ie, use of ≥2 different cannabis products) among adolescents has not been published, to our knowledge. Data elucidating whether similar trends might emerge with adolescent use of cannabis products are needed to guide surveillance, policy, and prevention addressing youth cannabis use.

In this cross-sectional survey, we report the prevalence, patterns, and sociodemographic correlates of adolescent cannabis use and polyuse across combustible, edible, and vaporized administration methods among 10th-grade students in Los Angeles, California, in 2015. This location and time are of significance; the state of California legalized medical cannabis in 1996 and recreational cannabis sales in 2018. Because Los Angeles is among the most populous and sociodemographically diverse metropolitan regions in the United States, cannabis use patterns among youths in this region can be informative of a wide cross-section of US youths.

## Methods

### Participants and Procedures

Data were drawn from the Happiness and Health Study, an ongoing prospective cohort study of students in 10 high schools in the Los Angeles metropolitan area. Details on study design and methods are available elsewhere (D.B. and A.M.L., unpublished data, completed July 2018).^11^ Schools were recruited based on their proximity to the research site and their representation of a diverse cross-section of urban and suburban communities, which collectively form a sociodemographically heterogeneous sample. Paper-and-pencil surveys were administered in students’ classrooms on site. Students not in class during data collections completed abbreviated surveys by telephone, internet, or mail. Survey items assessing cannabis use by method of administration were included in the (10th grade) survey from January 2 through October 6, 2015. Of 4100 eligible 9th-grade students in 2013, 3396 (82.8%) assented and consented to enroll. The institutional review board of the University of Southern California approved the study, which follows Strengthening the Reporting of Observational Studies in Epidemiology (STROBE) reporting guideline for cross-sectional studies^12^ and the American Association for Public Opinion Research (AAPOR) reporting guideline for surveys.^13^

Among 3251 participants administered the spring 2015 survey, 3177 provided cannabis use data, constituting this report’s analytic sample. The participant accrual diagram and comparisons of participant characteristics between cohort enrollees who were included vs excluded from this report due to attrition or missing responses can be found in the eFigure and eTable 1 in the Supplement. Cohort enrollees who were included vs excluded from this report differed on race/ethnicity, were slightly younger, and had lower proportions of boys and combustible cannabis users in the fall of 2013 (9th grade) (eTable 1 in the Supplement).

### Measures

#### Sociodemographic Characteristics

At enrollment, participants self-reported their sex, age, race/ethnicity (response options: white, black, Hispanic, Asian, Native American/Alaska Native, Native Hawaiian/Pacific Islander, multiethnic/multiracial, or other), parent with the highest educational attainment (6 choices ranging from 8th grade or less to advanced college degree), and eligibility for the free or subsidized lunch program (ie, youth with family income ≤185% of federal poverty limit [yes or no]). Respondents who selected Native American/Alaska Native (32 [1.0%]), Native Hawaiian/Pacific Islander (136 [4.3%]), or other (48 [1.5%]) for the race/ethnicity item were collapsed into a category of other owing to smaller sample sizes for these groups. To capture educational attainment and income in a socioeconomic status (SES) composite variable, we classified youths with high SES as those who reported their parents attended college or higher educational attainment (1946 [61.3%]) and being ineligible for free or subsidized lunch (1424 [44.8%]), as in prior work.^14,15^ Remaining participants were classified with low SES.

#### Cannabis Use

Similar to the Youth Risk Behavior Surveillance Survey^16^ and the Monitoring the Future Questionnaire,^17^ youths were asked, “Have you ever used the following substances in your life?” and “In the last 30 days, how many total days have you used...?” (response options: 0, 1-2, 3-5, 6-9, 10-19, 20-29, and 30 days) for each cannabis product. Cannabis items were worded as (1) “smoking marijuana (pot, weed, hash, reefer, or bud)” for combustible; (2) “marijuana or THC food or drinks (pot brownies, edibles, butter, oil)” for edible; and (3) *“*electronic device to vape liquid THC or hash oil (liquid pot, dabbing, weed pen)” for vaporized.

### Statistical Analysis

#### Descriptive Results and Comparisons by Method of Administration

Data were analyzed from July 17, 2017, through July 12, 2018. For each cannabis product and pattern of use and polyuse, descriptive frequencies (number [percentage]) were reported in the overall sample and by sex, race/ethnicity, and SES. We estimated multivariable generalized linear mixed models (GLMMs), including age, sex, SES, race/ethnicity, and method of cannabis administration as simultaneous regressors with random intercepts to account for school-clustering effects. Generalized linear mixed models allow the analysis of multiple observations nested within respondents, which permitted head-to-head comparisons in prevalence or frequency of use across the 3 products by inputting administration method (combustible vs edible vs vaporized) as a within-participant categorical regressor variable. To examine whether sociodemographic variation in use differed by cannabis administration method, within-by-between participant variable interaction terms were added (ie, administration method × sex, administration method × race/ethnicity, and administration method × SES) in subsequent models tested one at a time. Significant interaction effects were followed by stratum-specific univariable tests of associations of sociodemographic variables with cannabis use, completed separately for combustible, edible, and vaporized cannabis administration methods with school-level random effects.

Ever use and past 30-day use status (yes or no) were tested using binary logistic regression GLMMs with a binomial error distribution and log link. Frequency outcomes for past 30-day use were recoded into quantitative variables by inputting the mean whole number value within the range provided within each response category (range, 0-30), tested using linear regression GLMMs and including only the subsample of past 30-day users of cannabis products to characterize use frequency among users.

#### Polyuse

Polytomous (multinomial) logistic regression GLMMs^18^ with a generalized logit link, which allows for a categorical outcome with 2 or more levels, were used to test associations of each demographic variable with a nominal 4-group variable reflecting the number of cannabis products used (0, 1, 2, or 3) with school-level random effects and 1 observation per respondent. Separate models were tested for designations of ever and past 30-day use. Polytomous logistic regression provided an omnibus *P* value indicative of sociodemographic differences across any of the 4 groups and 3 pairwise contrast estimates comparing odds of use of 1, 2, or 3 methods of administration vs a reference outcome group who used none of the cannabis products.

Odds ratios (ORs) or unstandardized linear regression parameters (Β values) with 95% CIs from GLMMs are reported. Analyses were conducted using SPSS software (version 23; IBM Corp). Raw *P* values from 2-tailed tests of GLMMs were considered statistically significant after correction for multiple testing using the Benjamini-Hochberg method to control for the false discovery rate to .05, which led to a corrected significance threshold of .028.^19^

#### Missing Data

Sociodemographic data were unavailable for some participants due to item-level nonresponse or selecting “I don’t know” in response to SES questions (eTable 2 in the Supplement provides numbers with available data for each sociodemographic group). Participants with missing sociodemographic data were excluded from regression models (n = 476). Sensitivity analysis using multiple imputation with the missing-at-random assumption for sociodemographic data did not appreciably change results (eTable 3 in the Supplement).

## Results

### Participants

The sample of 3177 respondents was balanced by sex (1462 boys [46.0%] and 1715 girls [54.0%]), with a mean (SD) age of 16.1 (0.4) years. Respondents were diverse in race/ethnicity (537 [17.2%] Asian; 149 [4.8%] black; 1510 [48.3%] Hispanic; 507 [16.2%] white; 207 [6.6%] multiethnic/multiracial; and 216 [6.9%] other) and constituted a ratio of 60:40 of low (1654 [60.1%]) vs high (1099 [39.9%]) SES (eTable 2 in the Supplement).

### Cannabis Use Prevalence and Frequency by Method of Administration

Most cannabis users (665 [61.7%]) used multiple administration methods. As depicted in Table 1, prevalence of ever use was highest for combustible cannabis (993 [31.3%]), followed by edible (676 [21.3%]) and then vaporized (333 [10.5%]) cannabis. A similar pattern was found for past 30-day use (426 [13.4%] for combustible, 249 [7.8%] for edible, and 156 [4.9%] for vaporized cannabis). Depending on the time interval, odds of use were 3.21 to 4.15 times greater for combustible vs vaporized cannabis, 1.74 to 1.90 times as large for combustible vs edible cannabis, and 1.69 to 2.38 times greater for edible vs vaporized cannabis (Table 2). Among participants who reported using cannabis in the past 30 days, mean frequency of use of combustible cannabis was higher by 2.65 days (95% CI, 1.40-3.91 days; *P* = .001) than the mean frequency of use for edible cannabis and 1.75 days higher (95% CI, 0.59-2.90 days; *P* = .003) than frequency of use for vaporized cannabis. The difference in mean frequency of past-month use for edible vs vaporized cannabis (−0.91 days; 95% CI, −2.33 to 0.52 days; *P* = .21) was not significant (Table 2).

**Table 1.** Prevalence and Frequency Distribution of Cannabis Use by Administration Method in Overall Sample

Cannabis Use	Prevalence, No. (%) (n = 3177)						
	Any Cannabis Use	Administration Method			No. of Administration Methods Used		
		Combustible	Edible	Vaporized	1	2	3
Ever use	1077 (33.9)	993 (31.3)	676 (21.3)	333 (10.5)	412 (13.0)	405 (12.7)	260 (8.2)
Past 30-d use	474 (14.9)	426 (13.4)	249 (7.8)	156 (4.9)	216 (6.8)	159 (5.0)	99 (3.1)
Frequency of use among past 30-d users, d^a^							
1-2	NA	153 (35.9)	128 (51.4)	60 (38.5)	NA	NA	NA
3-5	NA	80 (18.8)	41 (16.5)	32 (20.5)	NA	NA	NA
6-9	NA	47 (11.0)	22 (8.8)	20 (12.8)	NA	NA	NA
10-19	NA	58 (13.6)	28 (11.2)	21 (13.5)	NA	NA	NA
20-29	NA	47 (11.0)	11 (4.4)	7 (4.5)	NA	NA	NA
All 30	NA	41 (9.6)	19 (7.6)	16 (10.3)	NA	NA	NA

Abbreviation: NA, not applicable.

^a^ The denominator of each column is the number of past 30-day cannabis users in each administration method.

**Table 2.** Differences in Cannabis Use and Sociodemographic Correlates of Cannabis Use by Administration Method

Regressor	Outcome					
	Ever Use^a^		Past 30-d Use^a^		Days Used in Past 30 d^b^	
	OR (95% CI)	*P* Value	OR (95% CI)	*P* Value	β (95% CI)	*P* Value
**Main Effect of Administration Method**^**c**^						
Combustible vs vaporized	4.15 (3.67 to 4.70)	<.001^d^	3.21 (2.83 to 3.63)	<.001^d^	1.75 (0.59 to 2.90)	.003^d^
Edible vs vaporized	2.38 (2.04 to 2.78)	<.001^d^	1.69 (1.28 to 2.22)	<.001^d^	−0.91 (−2.33 to 0.52)	.21
Combustible vs edible	1.74 (1.58 to 1.92)	<.001^d^	1.90 (1.56 to 2.31)	<.001^d^	2.65 (1.40 to 3.91)	.001^d^
**Sociodemographic Correlates of Use by Administration Method**						
Sex × administration method interaction estimate^c^^,^^e^	NA	.027^d^^,^^f^	NA	.003^d^^,^^f^	NA	.70^f^
Sex estimates stratified by administration method^f^						
Female vs male (outcome: combustible)	0.99 (0.85 to 1.16)	.96	0.93 (0.76 to 1.14)	.50	−3.68 (−5.53 to −1.83)	<.001^d^
Female vs male (outcome: edible)	1.08 (0.91 to 1.28)	.40	0.87 (0.67 to 1.12)	.28	−2.39 (−4.52 to −0.25)	.03
Female vs male (outcome: vaporized)	0.79 (0.63 to 0.99)	.04	0.62 (0.45 to 0.85)	.004^d^	−2.72 (−5.69 to 0.23)	.07
SES × administration method interaction estimate^c^^,^^e^	NA	<.001^d^^,^^f^	NA	<.001 ^d^^,^^f^	NA	.50^f^
SES estimates stratified by administration method^f^						
Low vs high SES (outcome: combustible)	1.71 (1.41 to 2.07)	<.001^d^	1.29 (1.01 to 1.67)	.04	−0.97 (−3.12 to 1.17)	.37
Low vs high SES (outcome: edible)	1.49 (1.20 to 1.84)	<.001^d^	1.19 (0.86 to 1.64)	.31	0.86 (−1.71 to 3.43)	.51
Low vs high SES (outcome: vaporized)	1.04 (0.79 to 1.38)	.77	0.91 (0.62 to 1.33)	.61	−0.76 (−4.10 to 2.58)	.65
Race/ethnicity × administration method interaction estimate^d^^,^^e^^,^^g^	NA	.49^f^	NA	.62^f^	NA	.44^f^

Abbreviations: NA, not applicable; OR, odds ratio; SES, socioeconomic status.

^a^ Binary logistic regression models in overall sample with data available for all regressors (n = 2701).

^b^ Linear regression among past 30-day users (n = 393).

^c^ Estimates from generalized linear mixed models of association of sociodemographic characteristics, administration method, and their interaction as simultaneous regressors, adjusted for school random effects and respondents’ age. Main effects of sociodemographic variables are not presented.

^d^ Indicates statistically significant with Benjamini-Hochberg correction using a *P* value threshold of .028 as the criterion of statistical significance.

^e^ Interaction terms were added to models one at a time; main effect estimates exclude interaction terms.

^f^ Estimates from univariable generalized linear mixed models with school random effects.

^g^ Stratum-specific associations of race/ethnicity with cannabis use for each administration method are reported in eTable 4 in the Supplement.

### Sociodemographic Correlates of Cannabis Use by Method of Administration

**Figure 1.**
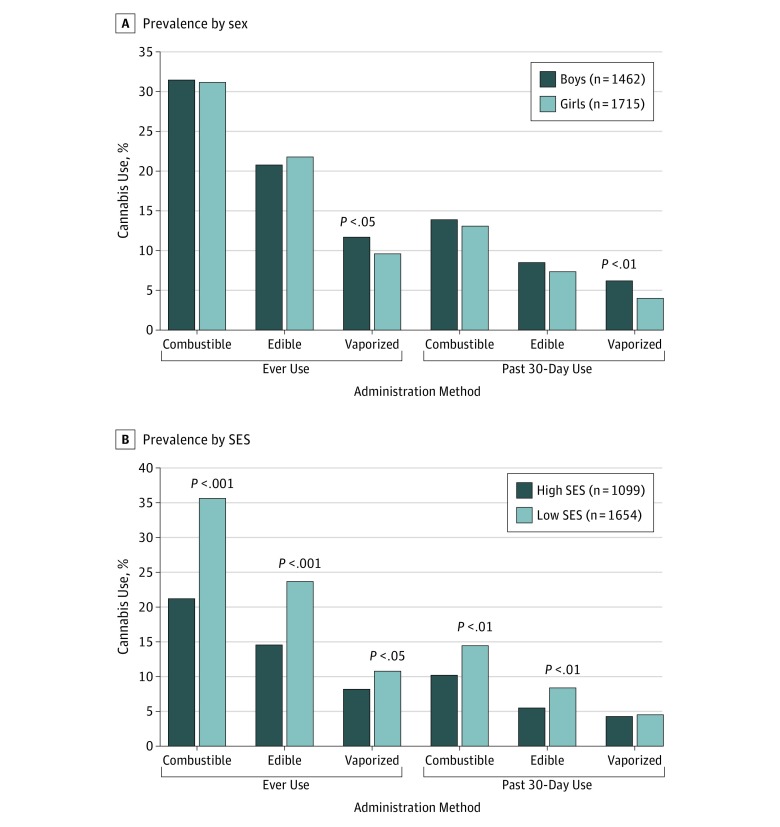
Prevalence of Combustible, Edible, and Vaporized Cannabis Product Use Prevalence is compared by sex (A) and socioeconomic status (SES) (B). Socioeconomic status was measured as high if at least 1 parent attended college and family income was greater than 185% of the federal poverty line (ineligible for free or subsidized lunch); low, if parental educational attainment was lower or family income was at or below 185% of the federal poverty line. *P* values were calculated using χ^2^ test to compare girls vs boys or low vs high SES.

As shown in Table 2, sex and SES differences in prevalence varied by administration method (administration method × sex and administration method × SES interactions) for ever use and past 30-day use. As illustrated in Table 2 and Figure 1, prevalence of ever use was significantly lower for girls than for boys for vaporized (163 [9.5%] vs 170 [11.6%]; OR, 0.79; 95% CI, 0.63-0.99; *P* = .04) but not combustible (534 [31.1%] vs 459 [31.4%]; OR, 0.99; 95% CI, 0.85-1.16; *P* = .96) or edible (373 [21.7%] vs 303 [20.7%]; OR, 1.08; 95% CI, 0.91-1.28; *P* = .40) cannabis. Socioeconomic differences in prevalence of ever use were significantly higher for low vs high SES for combustible (614 [37.1%] vs 242 [22.0%]; OR, 1.71; 95% CI, 1.41-2.07; *P* < .001) and edible (408 [24.7%] vs 166 [15.1%]; OR, 1.49; 95% CI, 1.20-1.84; *P* < .001) but not vaporized (186 [11.2%] vs 93 [8.5%]; OR, 1.04; 95% CI, 0.79-1.38; *P* = .77) cannabis. Differences in the association of SES and sex in past 30-day cannabis use status by administration method followed a similar pattern, eg, current use was higher in boys than girls for vaporized (89 [6.1%] vs 67 [3.0%]) but not for combustible (202 [13.8%] vs 224 [13.1%]) or edible (123 [8.4%] vs 126 [7.3%]) cannabis. Race/ethnicity differences in use status did not vary by method of administration for ever use (race/ethnicity × administration method) or past 30-day use (race/ethnicity × administration method) (stratum-specific associations between race/ethnicity and cannabis use for each administration method are reported in eTable 4 in the Supplement). Sociodemographic differences in frequency of use among past 30-day users did not vary by administration method.

### Prevalence and Sociodemographic Correlates of Polyuse of Cannabis Products

**Figure 2.**
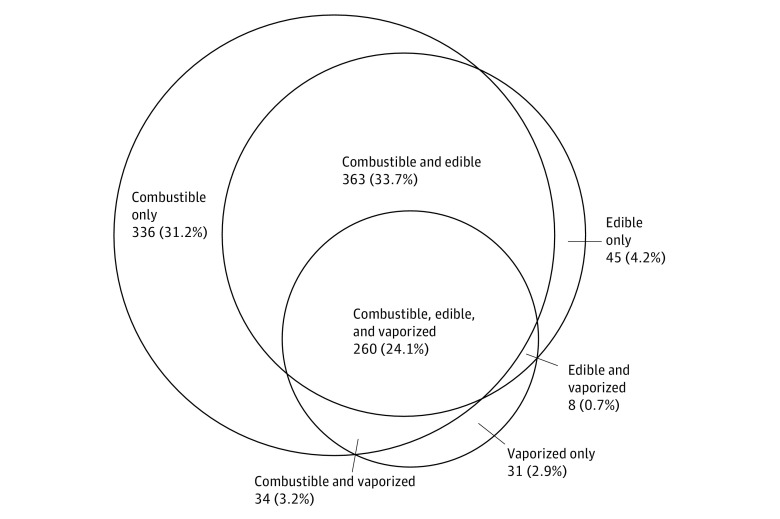
Prevalence of Ever Use Patterns of Single and Multiple Products in Cannabis Users Data are expressed as the number (percentage) of 1077 ever users of cannabis in any form. The size of the circles is proportional to the number of respondents in each group.

Dual-product cannabis use was reported by 405 (12.7%) youths reporting ever use and 159 (5.0%) reporting past 30-day use. Use of all 3 cannabis products was reported by 260 youths (8.2%) reporting ever use and 99 (3.1%) reporting past 30-day use. Among 1077 ever users of cannabis in any form, common patterns were dual ever use of combustible and edible products (363 [33.7%]), exclusive ever use of combustible cannabis (336 [31.2%]), and polyuse ever of all 3 administration methods (260 [24.1%]) (Figure 2). Among the 1077 ever users of cannabis, 84 (7.8%) never smoked combustible cannabis (31 [2.9%] were exclusive vaporized users; 45 [4.2%], exclusive edible users; and 8 [0.7%], edible and vaporized dual-product users).

In Table 3, youths with low (vs high) SES (OR, 1.63; 95% CI, 1.13-2.35) and black (OR, 2.47; 95% CI, 1.32-4.63), Hispanic (OR, 2.86; 95% CI, 1.86-4.40), white (OR, 2.04; 95% CI, 1.32-3.13), and multiethnic/multiracial (OR, 1.74; 95% CI, 1.27-2.38) (vs Asian) youths had greater odds of reporting polyuse ever of cannabis via 2 methods of administration (vs no use of any form of cannabis). With some exceptions, similar patterns of socioeconomic differences were also found for odds of triple-product polyuse involving ever use of all 3 cannabis administration methods (vs no use of cannabis) as well as polyuse of cannabis products in the past 30 days (eg, youths with low vs high SES [OR, 1.41; 95% CI, 1.06-1.87] had greater odds of reporting polyuse of cannabis products in the past 30 days) (Table 3).

**Table 3.** Association of Sociodemographic Characteristics With Use and Polyuse of Cannabis via Multiple Administration Methods

Regressor	Omnibus *P* Value	Odds Relative to No Use of Cannabis in Any Form, OR (95% CI)		
		Single-Product Use (1 Administration Method)	Dual-Product Use (2 Administration Methods)	Triple-Product Use (3 Administration Methods)
**Ever Use**^a^				
Female (vs male) sex	.24	0.99 (0.83-1.17)	1.13 (0.84-1.53)	0.79 (0.50-1.27)
Low (vs high) SES	<.001^b^	1.73 (1.40-2.14)^b^	1.63 (1.13-2.35)^b^	1.29 (1.02-1.62)
Race/ethnicity (vs Asian)				
Black	<.001^b^	3.35 (1.38-8.10)^b^	2.47 (1.32-4.63)^b^	3.72 (1.86-7.44)^b^
Hispanic		2.07 (1.42-3.03)^b^	2.86 (1.86-4.40)^b^	5.61 (3.16-9.96)^b^
White		1.55 (0.78-3.08)	2.04 (1.32-3.13)^b^	4.70 (2.61-8.47)^b^
Multiethnic/multiracial		1.53 (0.91-2.58)	1.74 (1.27-2.38)^b^	6.16 (3.21-11.81)^b^
Other		2.32 (1.30-4.17)^b^	1.65 (0.99-2.73)	5.81 (3.27-10.31)^b^
**Past 30-Day Use**^a^				
Female (vs male) sex	<.001^b^	1.33 (1.07-1.65)^b^	1.30 (0.76-2.24)	0.37 (0.19-0.71)^b^
Low (vs high) SES	.07	1.24 (0.81-1.91)	1.41 (1.06-1.87)^b^	1.05 (0.59-1.88)
Race/ethnicity (vs Asian)				
Black	<.001^b^	1.88 (0.50-7.10)	3.18 (1.25-8.09)^b^	8.98 (1.98-40.72)^b^
Hispanic		1.62 (0.81-3.25)	4.07 (1.87-8.87)^b^	7.69 (2.34-25.26)^b^
White		2.00 (0.93-4.27)	3.34 (1.36-8.23)^b^	11.20 (3.08-40.68)^b^
Multiethnic/multiracial		2.47 (1.05-5.79)	3.02 (1.03-8.87)	10.68 (2.64-43.18)^b^
Other		1.42 (0.63-3.20)	3.54 (1.32-9.47)^b^	7.62 (2.13-27.26)^b^

Abbreviations: OR, odds ratio; SES, socioeconomic status.

^a^ From polytomous logistic regression models including demographic characteristics as simultaneous regressors and school random effects adjusted by age (n = 2701).

^b^ Indicates statistically significant with Benjamini-Hochberg correction using a *P* value threshold of .028 as the criterion of statistical significance.

## Discussion

This study of Los Angeles 10th-grade students in 2015 found that, although combustible cannabis remained the most popular cannabis administration method, use of cannabis via alternative administration methods was of appreciable prevalence and unequally distributed across sociodemographic strata. We found that polyuse via multiple administration methods was a predominant pattern of cannabis use and report the first evidence, to our knowledge, of triple-product polyuse of combustible, edible, and vaporized cannabis among youths. These results add to a scant evidence base and begin to address critical early questions about the potential effect of alternative cannabis products on the pediatric public health burden.

A key question is whether a new pool of youth who have traditionally been at lower risk for combustible cannabis uptake might be drawn to alternative (noncombustible) cannabis products.^7^ Edible and vaporized cannabis possess unique qualities absent in combustible cannabis (eg, availability of flavorings, no smell of smoke, attractive marketing and packaging, absence of harsh sensations to the airways and bitter taste of smoke), which might attract a wider segment of youths normally deterred from cannabis in combustible form.^20,21^ Although this study does not definitively address this question, observations from the present analysis fail to dispel such concerns. Among ever users of cannabis in this study, 7.8% had never smoked combustible cannabis, having used only edible or vaporized products. Whether this group of exclusive edible or vaporized cannabis product users would have avoided cannabis use if noncombustible products were unavailable is unknown.

Differences in cross-product frequency of use in this sample provide some of the first observational data indicative of the comparative abuse liability of combustible, edible, and vaporized cannabis in adolescents. Mean frequency of cannabis use was higher for combustible than edible cannabis. Evidence suggested that frequency of vaping was at the midpoint for the frequency observed for combustible and edible product use. Some evidence in adults shows that desirable psychoactive effects are fairly reliable with combustible cannabis but can be lower or less reliable with vaporized and edible cannabis, owing in part to the variable pharmacokinetics of cannabinoid delivery with these methods and greater product diversity that might affect drug delivery.^22,23,24^

Recent surveys of adolescent Facebook users and northern California residents^8,9^ suggest that noncombustible products appear to be commonly used in addition to combustible products, with polyuse of cannabis products being a predominant use pattern. The present study reinforces this conclusion and reveals a novel pattern of triple-product use of combustible, edible, and vaporized cannabis products, which represented 260 of 1077 cases (24.1%) of ever use and 99 of 474 (20.9%) of past 30-day use of cannabis in this sample. Relative to single-product use, polyuse of cannabis products might increase neuroexposure to a wider diversity or higher level of cannabinoids, which could accelerate the risks associated with cannabis use, including dependence and other neurocognitive consequences.^25^ Given such potential health hazards and the sizeable prevalence of dual- and triple-product cannabis use in this study, national surveillance of patterns of polyuse of cannabis products is warranted. With impending expansion of the cannabis product marketplace in states that recently passed legal recreational cannabis legislation, surveillance might be an urgent need.

The present data suggest that the emergence of alternative cannabis products might redefine populations at risk and the potential for sociodemographic disparities in cannabis use. Sex differences in use were more pronounced for vaporized (vs edible or combustible) cannabis, suggesting that this administration method might differentially appeal to male users and/or deter female users. Socioeconomic disparities in use were characteristically observed for combustible and edible cannabis, yet virtually absent for vaporized products, similar to recently reported evidence of associations of low SES with combustible but not vaporized tobacco product use in Connecticut youth.^26^ Whether the comparatively high initial cost of vaporized cannabis products is a financial barrier that prohibits access in teenagers with lower SES, certain features of vaporized products disproportionately appeal to higher-SES youths, or other factors explain this trend warrant future study. Considerably higher prevalence of polyuse of cannabis products was observed for lower (vs higher) SES, boys (vs girls), and certain racial/ethnic groups. Given these results, cannabis regulatory policies or prevention programs that target particular products might have an uneven effect on rates of cannabis use across different demographic subgroups.

### Limitations

This study had several limitations. First, because this report was cross-sectional, whether youth polyusers first initiated cannabis use with noncombustible products and later transitioned to combustible cannabis or vice versa is unclear, which can be addressed in future longitudinal work. Second, study survey items did not differentiate cannabis product potency, strain (sativa vs indica vs hybrid), or certain types of cannabis formulation used, including the use of waxes, topicals, tinctures, concentrates, and cannabis-tobacco mixtures. These distinctions might be important because use of high-potency cannabis concentrates, especially via vaporized administration methods, appears to be popular among adult cannabis users and is associated with living in states with recreational cannabis policies, younger age, and lower perceived risk of cannabis use.^27^ Third, specific features of the sampling design (eg, a convenience sample in 2015 of 10th-grade students from a single region with a high prevalence of racial and ethnic minorities, attrition before the 10th grade survey) may have introduced selection bias, and results may not generalize to the current year or across historical periods, locations, race/ethnicity, or age ranges. Fourth, this study used a measure of SES based on parents’ educational attainment and the students’ eligibility for free or subsidized lunch, which may not generalize to other SES indicators. Fifth, although we assumed that data are missing at random conditional on the values of variables used for multiple imputation,^28^ we cannot rule out potential bias due to missingness caused by unmeasured aspects of respondents’ sociodemographic characteristics. Sixth, relative to youth in this report, those excluded owing to cohort attrition before the spring 2015 survey or other sources of missing data (219 of 3396 [6.4%] cohort enrollees) were slightly overrepresented by boys, older youths, certain racial/ethnic groups, and combustible cannabis users based on data collected in 9th grade. Consequently, the figures reported herein might underestimate cannabis use.

## Conclusions

In this 2015 cross-sectional survey of Los Angeles 10th-grade students, use of cannabis via alternative administration methods was of appreciable prevalence, predominantly observed in conjunction with polyuse of other cannabis products, and unequally distributed across sociodemographic strata. Surveillance, regulatory restrictions, and prevention of adolescent use across the increasingly diverse spectrum of cannabis products might be warranted to manage the cannabis-related pediatric public health burden.

## Notes

Supplement. **eTable 1.** Differences Between Study Enrollees Included (n = 3177) vs Excluded (n = 219) in the Analytic Sample on Participant Characteristics at Study Enrollment (Fall 9th Grade) **eTable 2.** Prevalence of Cannabis Use by Administration Method and Demographic Characteristics **eTable 3.** Differences in Cannabis Use and Sociodemographic Correlates of Cannabis Use by Administration Method Using Multiple Imputation to Address Missing Sociodemographic Data (n = 3177) **eTable 4.** Association of Race/Ethnicity With Cannabis Use, Stratified by Administration Method **eFigure.** Recruitment Flowchart
