# Supplementary material for: Prevalence and Sociodemographic Correlates of Adolescent Use and Polyuse of Combustible, Vaporized, and Edible Cannabis Products
Source: JAMA Netw Open. 2018 Sep 28;1(5):e182765. doi: 10.1001/jamanetworkopen.2018.2765 (PMC6324616; doi:10.1001/jamanetworkopen.2018.2765)
Supplement: Supplement. — eTable 1. Differences Between Study Enrollees Included (n = 3177) vs Excluded (n = 219) in the Analytic Sample on Participant Characteristics at Study Enrollment (Fall 9th Grade) eTable 2. Prevalence of Cannabis Use by Administration Method and Demographic Characteristics eTable 3. Differences in Cannabis Use and Sociodemographic Correlates of Cannabis Use by Administration Method Using Multiple Imputation to Address Missing Sociodemographic Data (n = 3177) eTable 4. Association of Race/Ethnicity With Cannabis Use, Stratified by Administration Method eFigure. Recruitment Flowchart [file jamanetwopen-1-e182765-s001.pdf]

Supplementary Online Content

Peters EN, Bae D, Barrington-Trimis JL, Jarvis BP, Leventhal AM. Prevalence and sociodemographic correlates of adolescent use and polyuse of combustible, vaporized, and edible cannabis products. *JAMA Netw Open.* 2018;1(5):e182765. doi:10.1001/jamanetworkopen.2018.2765

eTable 1. Differences Between Study Enrollees Included (n = 3177) vs Excluded (n = 219) in the Analytic Sample on Participant Characteristics at Study Enrollment (Fall 9th Grade)

eTable 2. Prevalence of Cannabis Use by Administration Method and Demographic Characteristics

eTable 3. Differences in Cannabis Use and Sociodemographic Correlates of Cannabis Use by Administration Method Using Multiple Imputation to Address Missing Sociodemographic Data (n = 3177)

eTable 4. Association of Race/Ethnicity With Cannabis Use, Stratified by Administration Method

eFigure. Recruitment Flowchart

This supplementary material has been provided by the authors to give readers additional information about their work.

©2018 Peters EN et al. *JAMA Network Open.*

eTable 1. Differences Between Study Enrollees Included (n = 3177) vs Excluded (n = 219) in the Analytic Sample on Participant Characteristics at Study Enrollment (Fall 9th Grade)

	Included (N = 3177)		Excluded (N = 219)	*P* Value
	No. (%) or Mean (SD)		No. (%) or Mean (SD)	
Sex				
Available sample, No.	3177		217	
Male, No. (%)	1462 (46.0)		119 (54.8)	.01
Female, No. (%)	1715 (54.0)		98 (45.2)	
Age, Mean (SD)	14.57 (.40)		14.63 (.43)	.05
Available sample, No.	3175		204	
Race/ethnicity				
Available sample, No.	3126		200	
Asian, No. (%)	537 (17.2)		23 (11.5)	.03
African American, No. (%)	149 (4.8)		17 (8.5)	
Hispanic, No. (%)	1510 (48.3)		95 (47.5)	
White, No. (%)	507 (16.2)		37 (18.5)	
Multiethnic or multiracial, No. (%)	207 (6.6)		18 (9.0)	
Other,[a] No. (%)	216 (6.8)		10 (5.0)	
**Combustible cannabis use**				
Ever use, No. (%)	463 (14.7)		68 (33.8)	<.001
Available sample, No.	3147		201	
Past 30-day use, No. (%)	235 (7.4)		36 (17.8)	<.001
Available sample, No.	3164		202	

Note. [a]Other category combines American Indian/Alaska Native, Native Hawaiian/Pacific Islander, and respondents who did not self-identify with any of the categories provided.

©2018 Peters EN et al. *JAMA Network Open.*

eTable 2. Prevalence of Cannabis Use by Administration Method and Demographic Characteristics[a]

	Sex		Race/Ethnicity						SES	
	**Males (n=1462)[c]**	**Females (n=1715)[c]**	**Asian (n=537)[c]**	**Black (n=149)[c]**	**Hispanic (n=1510)[c]**	**White (n=507)[c]**	**Multiethnic (n=207)[c]**	**Other[b] (n=216)[c]**	**High (n=1099)[c]**	**Low (n=1654)[c]**
Ever use										
Combustible	459 (31.4)	534 (31.1)	77 (14.3)	52 (34.9)	575 (38.1)	140 (27.6)	63 (30.4)	62 (28.7)	242 (22.0)	614 (37.1)
Edible	303 (20.7)	373 (21.7)	49 (9.1)	29 (19.5)	409 (27.1)	92 (18.1)	43 (20.8)	40 (18.5)	166 (15.1)	408 (24.7)
Vaped	170 (11.6)	163 (9.5)	16 (3.0)	15 (10.1)	189 (12.5)	50 (9.9)	29 (14.0)	27 (12.5)	93 (8.5)	186 (11.2)
Past 30 day use										
Combustible	202 (13.8)	224 (13.1)	27 (5.0)	21 (14.1)	238 (15.8)	77 (15.2)	33 (15.9)	21 (9.7)	116 (10.6)	248 (15.0)
Edible	123 (8.4)	126 (7.3)	13 (2.4)	12 (8.1)	148 (9.8)	38 (7.5)	17 (8.2)	15(6.9)	63 (5.7)	144 (8.7)
Vaped	89 (6.1)	67 (3.9)	6 (1.1)	6 (4.0)	83 (5.5)	33 (6.5)	11 (5.3)	13(6.0)	49 (4.5)	78 (4.7)

[a]Data are expressed as No.(%). [b]Other category combines American Indian/Alaska Native (n = 32, 1.0%), Native Hawaiian/Pacific Islander (n = 136, 4.4%), and respondents who did not self-identify with any of the categories provided (n = 48, 1.5%). [c]The denominator is for each column.

©2018 Peters EN et al. *JAMA Network Open.*

eTable 3. Differences in Cannabis Use and Sociodemographic Correlates of Cannabis Use by Administration Method Using Multiple Imputation to Address Missing Sociodemographic Data (n = 3177)

	**Outcome[a]**					
	**Ever use[b]**		**Past 30-day use[b]**		**Days used in past 30 days[c]**	
**Regressor**	**OR (95% CI)**	***P* Value**	**OR (95% CI)**	***P* Value**	**B (95% CI)**	***P* Value**
**Main Effect of Administration Method[a]**						
Combustible vs. Vaped	4.11 (3.62 to 4.66)	<.001	3.10 (2.77 to 3.46)	<.001	1.71 (1.01 to 2.41)	<.001
Edible vs. Vaped	2.37 (2.05 to 2.73)	<.001	1.66 (1.33 to 2.08)	<.001	-1.00 (-1.76 to - -0.24)	.01
Combustible vs. Edible	1.74 (1.58 to 1.91)	<.001	1.86 (1.74 to 1.99)	<.001	2.71 (2.12 to 3.30)	<.001
**Sociodemographic Correlates of Use by Administration Method**	N/A	.01[e]	N/A	<.001[e]	N/A	.19[e]
Sex × Administration Method Interaction Estimate[a,d]						
Sex estimates stratified by administration method[e]	1.00 (0.78 to 1.27)	.98	0.93 (0.63 to 1.38)	.72	-3.49 (-4.26 to -2.71)	<.001
Female vs. Male (Outcome: Combustible)	1.08 (0.83 to 1.40)	.57	0.87 (0.60 to 1.26)	.45	-2.09 (-2.97 to -1.21)	<.001
Female vs. Male (Outcome: Edible)	0.79 (0.72 to 0.87)	<.001	0.62 (0.41 to 0.94)	.02	-2.64 (-3.83 to -1.44)	<.001
Female vs. Male (Outcome: Vaped)	N/A	<.001[e]	N/A	<.001[e]	N/A	.06[e]
SES × Administration Method Interaction Estimate[a,d]						
SES estimates stratified by administration method[e]	1.57 (1.46 to 1.69)	<.001	1.29 (1.14 to 1.38)	<.001	-1.40 (-2.27 to -0.53)	.002
Low vs. High SES (Outcome: Combustible)	1.34 (1.24 to 1.45)	<.001	1.13 (0.99 to 1.28)	.06	1.05 (0.03 to 2.07)	.04
Low vs. High SES (Outcome: Edible)	1.02 (0.91 to 1.13)	.78	0.85 (0.70 to 1.04)	.11	-0.01 (-1.34 to 1.30)	.98
Low vs. High SES (Outcome: Vaped)	N/A	.45[e]	N/A	.60[e]	N/A	.75[e]

Note. [a]Estimates from generalized linear mixed models (GLMM) of association of sociodemographics, administration method, and their interaction as simultaneous regressors, adjusted for school random effects and respondents' age. Main effects sociodemographic variables not presented. [b]Binary logistic regression models in overall sample (N = 3177). [c]Linear regression amongst past 30-day users (N = 474). [d]Interaction terms were added to models one at a time; main effect estimates exclude interaction terms. [e]Estimates from univariable generalized linear mixed models with school random effects.

©2018 Peters EN et al. *JAMA Network Open.*

eTable 4. **Association of Race/Ethnicity With Cannabis Use, Stratified by Administration Method**

	Outcome[a]							
	Ever use[b]			Past 30-day use[b]			Days used in past 30 days[c]	
**Regressor**	**OR (95% CI)**	***P* Value**		**OR (95% CI)**	***P* Value**		**b (95% CI)**	***P* Value**
**Outcome: Combustible**								
Asian (Reference)								
Black	2.35 (1.53 to 3.60)	<.001		2.33 (1.26 to 4.29)	.01		4.37 (-1.28 to 10.02)	.13
Hispanic	2.51 (1.87 to 3.36)	<.001		2.33 (1.51 to 3.62)	<.001		3.13 (-0.81 to 7.07)	.12
White	1.81 (1.28 to 2.55)	.001		2.27 (1.39 to 3.70)	.001		5.15 (0.81 to 9.49)	.02
Multiethnic	2.05 (1.38 to 3.04)	<.001		2.63 (1.52 to 4.54)	.001		3.31 (-1.73 to 8.35)	.20
Other	1.91 (1.29 to 2.83)	.001		1.54 (0.84 to 2.80)	.16		5.09 (-0.56 to 10.73)	.08
**Outcome: Vaporized**								
Asian (Reference)								
Black	1.93 (1.15 to 3.23)	.01		2.82 (1.25 to 6.37)	.01		3.81 (-3.14 to 10.76)	.28
Hispanic	2.73 (1.94 to 3.86)	<.001		3.15 (1.72 to 5.75)	<.001		2.02 (-3.12 to 7.16)	.44
White	1.99 (1.33 to 2.97)	.001		2.89 (1.47 to 5.65)	.002		0.25 (-5.47 to 5.97)	.93
Multiethnic	2.20 (1.39 to 3.48)	.001		2.98 (1.41 to 6.31)	.004		2.40 (-4.00 to 8.80)	.46
Other	1.89 (1.19 to 3.01)	.01		2.48 (1.16 to 5.33)	.02		1.33 (-5.31 to 7.97)	.69
**Outcome: Edible**								
Asian (Reference)								
Black	2.66 (1.27 to 5.57)	.01		3.42 (1.08 to 10.77)	.04		2.50 (-8.17 to 13.17)	.64
Hispanic	3.14 (1.82 to 5.42)	<.001		4.41 (1.89 to 10.31)	.001		-0.95 (-8.76 to 6.87)	.81

©2018 Peters EN et al. *JAMA Network Open.*

White	2.92 (1.59 to 5.36)	.001		5.51 (2.25 to 13.53)	<.001		-0.80 (-9.01 to 7.40)	.85
Multiethnic	4.09 (2.15 to 7.78)	<.001		4.48 (1.63 to 12.29)	.004		-0.50 (-9.88 to 8.88)	.92
Other	3.62 (1.90 to 6.91)	<.001		5.16 (1.94 to 13.73)	.001		-0.96 (-10.08 to 8.16)	.84

Note. [a]Estimates from univariable generalized linear mixed models with school random effects. [b]Binary logistic regression models in overall sample with data available for race/ethnicity (N = 3126). [c]Linear regression amongst past 30-day users (N = 464).

©2018 Peters EN et al. *JAMA Network Open.*

eFigure. Recruitment Flowchart

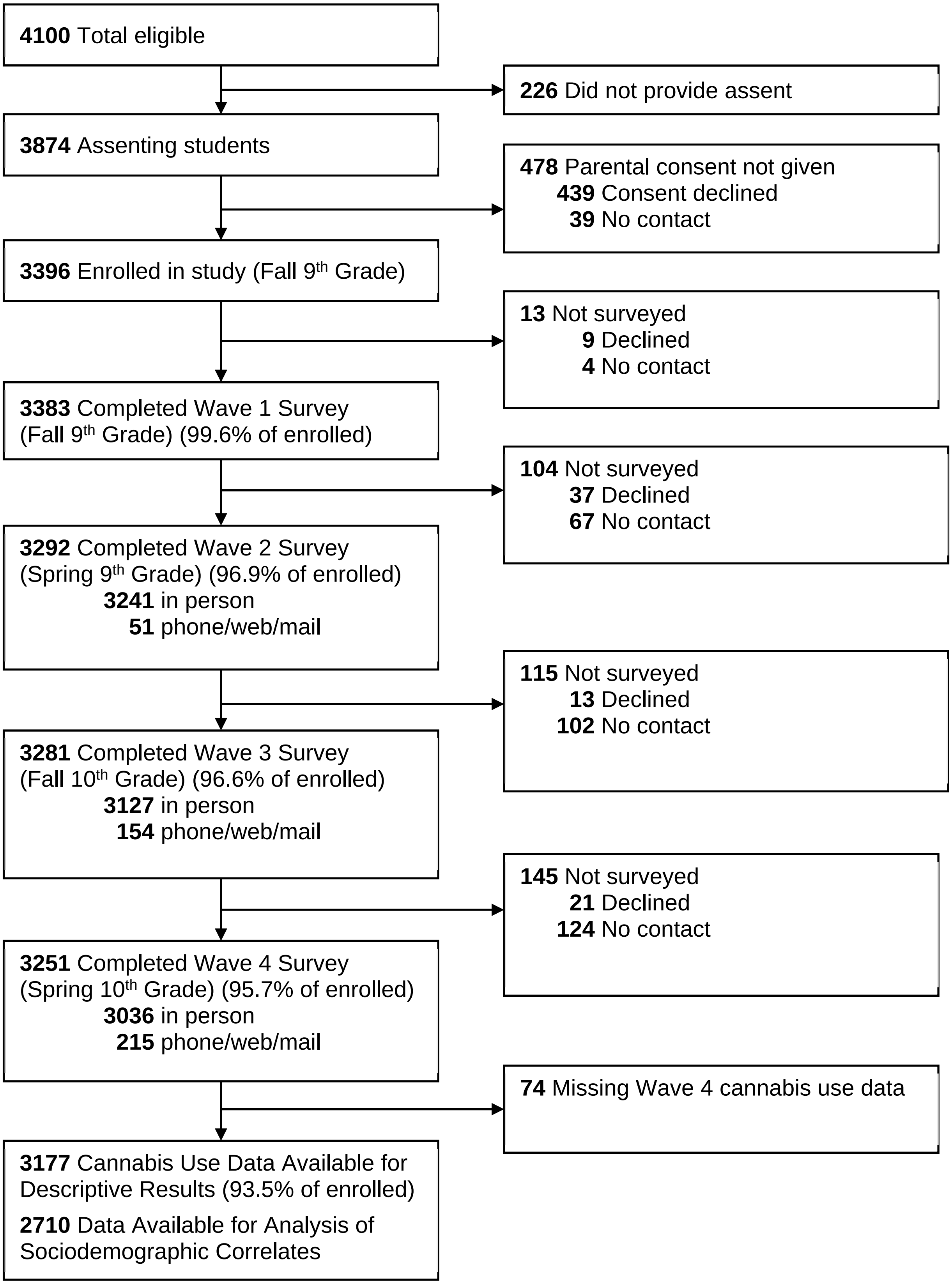

©2018 Peters EN et al. *JAMA Network Open.*
